# Empathy and mentalization as mediators between childhood maltreatment and social decision-making during adulthood

**DOI:** 10.1038/s41598-026-37273-9

**Published:** 2026-02-14

**Authors:** Steffi Benoit, Julie Maheux, Dominick Gamache, Sébastien Hétu

**Affiliations:** 1 Département de psychologie, Université du Québec à Trois-Rivières, Université du Québec à Trois-Rivières, Pavillon Michel-Sarrazin, rue Sainte-Marguerite, Local, Trois-Rivières, QC 3600, 2008, G9A 5H7 Canada; 2 CERVO Brain Research Center, CERVO Brain Research Centre, Chemin de la Canardière, Québec, QC 2601, G1J 2G3 Canada; 3 CRIPCAS Research Center, Département de psychologie de l’Université de Montréal Montréal, H3C3J7 Québec, QC Canada; 4 Département de psychologie, Université de Montréal, Université de Montréal, 90, avenue Vincent-D’Indy, Montreal, QC H2V 2S9 Canada; 5 CIRCA Research Center, CIRCA – Université de Montréal, Pavillon, Jean-Brillant, Bureau, Montréal, QC 3744, 260-15, H3C 3J7 Canada; 6 Département de Psychologie, Université de Montréal, Université de Montréal, Pav. Marie-Victorin, Montréal, Qc H2V 2S9 Canada

**Keywords:** Child maltreatment, Social decision-making, Mentalization, Empathy, Economic games, Neuroscience, Psychology, Psychology

## Abstract

Childhood maltreatment is a highly prevalent form of early adversity with long-lasting consequences for social and emotional functioning. While previous work has established links between maltreatment, empathy, and mentalization, little is known about how these processes shape adult social decision-making. In this study, 327 adults completed validated self-report measures of childhood maltreatment, empathy, and mentalization, along with a series of economic games designed to assess cooperation and punishment. We found that greater severity of childhood maltreatment was selectively associated with two decision-making patterns: reduced cooperation when observed by a third party and increased punishment of cooperative partners, a paradoxical behavior known as antisocial punishment. Mediation analyses revealed that these associations were explained by affective components of empathy. Specifically, diminished affective resonance (congruent emotional responses) mediated the link between maltreatment and reduced cooperation, while heightened affective dissonance (incongruent emotional responses) mediated the link between maltreatment and antisocial punishment. Mentalization did not uniquely mediate these relationships when considered alongside empathy. These findings highlight empathy disruptions as a key pathway through which early adversity may shape adult social behavior. By identifying specific affective mechanisms, this work points to potential targets for interventions aimed at improving social functioning in individuals with histories of maltreatment.

**Supplementary Information:**

The online version contains supplementary material available at 10.1038/s41598-026-37273-9.

## Introduction

Social decision-making shapes how we navigate everyday life. We continuously adjust our behavior across social settings, guided by updated information from our environment^1^. This process involves a range of skills, with empathy and mentalization playing particularly central roles^2,3^. Early social experiences are critical for the development of theses capacities^4,5^. While the long-lasting effects of childhood maltreatment (i.e., abuse and/or neglect) on empathy and mentalization have been documented (e.g.,^6,7^), much less attention has been given to its latent impact on social decision-making. Given that approximately one in four adults worldwide report having experienced childhood abuse and/or neglect^8^, several important, yet under-explored, questions arise: To what extent does having experienced childhood maltreatment influences one’s ability to make social decisions as an adult? And how might processes like empathy and mentalization, often disrupted by childhood relational trauma, mediate this relationship? The present study aims to assess childhood maltreatment severity, empathy, mentalization as well as social decision-making in an adult community sample, updating existing knowledge about the relationships between these variables and investigating how childhood maltreatment may influence social decision-making years later.

### Child maltreatment and developmental roots of empathy and mentalization

Child maltreatment ranks among the most detrimental experiences a person can face during the critical developmental period of childhood^9^. According to the World Health Organization^9^, “child maltreatment” refers to any form of physical or emotional abuse and/or neglect that undermines a child’s development, dignity, life, or health. Its global prevalence is striking, with nearly a billion children affected each year^10^ including sexual abuse (7.6% of boys, 18% of girls), physical abuse (22.6%), emotional abuse (36.3%), physical neglect (16.3%), and emotional neglect (18.4%)^11^. The consequences of childhood maltreatment are severe and long-lasting. Often involving multiple, overlapping forms, maltreatment can be chronic and disrupt emotional, cognitive, and psychosocial development^12^ and heighten the risk of re-victimization in later relationships^13^. Indeed, maltreatment typically occurs within a child’s most significant relationships during key periods for the development of identity, personality and of important socio-cognitive skills such as empathy and mentalization^5,14,15^.

#### *Empathy*

Empathy is a psychological process involving a combination of cognitive and affective skills, elicited by exposure to another person’s affective experience. Cognitive empathy refers to the ability to recognize and understand other’s feelings (e.g., I can recognize when someone is sad), while affective empathy involves an emotional response that can either be congruent with the other’s emotion (affective resonance, e.g., I get upset when I see someone in pain) or incongruent with it (affective dissonance, e.g., I get amused when I see someone crying^16^). Affective dissonance is conceptually analogous to *Schadenfreude*—the pleasure derived from another’s misfortune^17^.

Empathy is observed in children as early as six to eight months of age^15^ and in, simpler forms, from as early as three months of age or even in newborns (see^18^). Like most psychological processes, it emerges from a complex interaction between a child’s biological/genetic predispositions and their environment^4^. Developmental research highlights the critical role of caregiver sensitivity and parenting style in fostering this ability^4,19–22^. Empathy develops progressively within the experience of intersubjectivity and reciprocity between a child and their caregiver, laying the foundations for future social relationships^15,23,24^. When these early exchanges are characterized by maltreatment, the child’s capacity to detect, understand, and share emotional signals can be disrupted, impairing the developmental pathways that normally support empathy^23,25^. In their scoping review, Kahhale and colleagues (2025)^26^ report that most studies indicate a negative association between early life adversity—including child maltreatment—and cognitive empathy, whereas findings for affective empathy are mixed with negative, positive, and null associations reported in the literature.

This pattern aligns with meta-analytic evidence showing an overall association between childhood maltreatment and deficits in cognitive empathy, as well as in affective empathy, with the latter depending on the type of measure used to assess affective empathy^6^. Importantly, most studies have focused on the empathic resonance component of affective empathy, leaving the relationship between child maltreatment and empathic dissonance largely unexplored. Another socio-cognitive skill that seems to be affected by child maltreatment is mentalization.

#### *Mentalization*

Mentalization is defined as the ability to understand actions—both one’s own and others’—as meaningful and intentional, by recognizing that they are driven by underlying emotions, desires, goals, and beliefs unique to the individual^27–30^. Empathy and mentalization can be differentiated in terms of both their functions and the nature of the emotional information they process. Functionally, cognitive empathy refers to the identification of another person’s emotional state, whereas affective empathy concerns emotional reactions to others’ emotions^16^. In contrast, mentalization involves inferring plausible causes and underlying meanings of emotional states^29^.

These constructs also differ in the nature of the emotional information they process. Cognitive and affective empathy respectively focus on other’s emotions and on one’s own emotional reactions to others’ emotions^16^, whereas mentalization encompasses understanding and reacting to both others’ and one’s own emotions^29^. Importantly, unlike affective empathy—which can involve affective dissonance^16^—mentalization does not formally distinguish between incongruent and congruent emotional responses^29^.

Although these capacities may often align and complement one another, they remain partially overlapping yet clearly distinct social–cognitive skills^29,31,32^.

Mentalization also develops early in childhood, shaped through the attachment bond between a child and their caregivers^5^. Initially, young children operate in a pre-mentalizing mode, where they cannot yet distinguish between their internal experiences and the external world^5^. Over time, and within a secure attachment relationship that fosters emotional exploration, mentalization abilities begin to emerge, typically around the ages of four or five^33,34^. In the context of abuse or neglect, however, this developmental trajectory can be disrupted. Children may struggle to differentiate their own feelings from those of others, having not yet integrated the early building blocks of mentalization. As a result, they may experience difficulties in understanding the intentions and emotions of those around them^35^. Empirical research supports these links, showing that childhood maltreatment is associated with poorer mentalizing skills both during childhood and into adulthood, with more severe maltreatment predicting poorer mentalization skills^7,36^.

Overall, as complementary socio-cognitive skills, empathy and mentalization—both highly sensitive to early developmental experiences—are critical for appropriate interpersonal functioning. Variations in these capacities can, in turn, shape how individuals navigate social decision-making.

### The interplay between childhood adversity, social decision-making, empathy and mentalization

#### ***Social dilemmas: the landscape of social decision-making***

Social decision-making refers to the way we use information from our social environment to guide how we behave in interpersonal situations^1^. Most of our daily decisions are guided by interactions with our social environment^37^. To navigate these interactions effectively, we rely on psychological processes such as mentalization and empathy. These capacities help us detect, interpret, and respond to social cues in ways that are contextually appropriate^3^.

Social decision-making is often studied using simple ‘games’ (or social dilemmas) in which participants are faced with decisions that pit their personal interests against those of others, while facing uncertainty about other players’ choices. These games, derived from “Game Theory”^38^, offer ecologically valid measures of social decision-making and allow behaviors to be categorized by both their outcomes (e.g., cooperation or punishment) and underlying motivations^37^. For example, a player who sacrifices part of their resources to share with another player adopts a cooperative approach, while one who spends resources to remove those of another player adopts a punitive approach. Notably, both cooperation and punishment can reflect prosocial motives. Motivations for prosocial behavior include altruistically motivated actions (spending resources to benefit someone else), strategically motivated actions (spending resources based on cost–benefit calculations, such as expecting reciprocity), or norm-motivated actions (spending resources to comply or using punishment to enforce social norms^39^). Conversely, antisocial behaviors can also arise, such as antisocial punishment (spending resources to remove those of a prosocial player or to punish prosocial behavior) for which motives remain less understood but have been linked to envy, competition, or spite^40^. Building on this framework, Peysakhovich et al.^41^ identified consistent individual tendencies in these games, introducing the concepts of a cooperative phenotype (a stable preference for altruistically motivated cooperation). Their data also suggest a punitive phenotype (a stable tendency to engage in norm-enforcing punishment). These could reflect distinct motivational profiles underlying social decision-making.

#### ***When adversity shapes social preferences and decision-making***

Through our life experiences, we build inferences about the social world that shape our expectations toward others and guide how we make social decisions^42^. Adverse experiences, particularly those involving interpersonal trauma, can significantly alter social behavior—though not always in predictable ways.

The formative nature of childhood is particularly relevant in this regard. Early relational experiences play a central role in shaping long-term social functioning. For example, a 40-year longitudinal study by Luo et al.^43^ showed that adults who had participated in early childhood educational enrichment programs later displayed significantly more prosocial behavior in economic games than controls. Conversely, other longitudinal research highlights the long-term negative impact of early adversity: Raby et al.^44^ found that abuse and neglect in the first five years of life predicted poorer social competence in both peer and romantic relationships up to age 34, and Degli Esposti et al.^45^ documented a robust, persistent association between childhood maltreatment and antisocial behavior across the life course. Meta-analyses reinforce these findings, showing that childhood maltreatment is positively associated with antisocial behaviors (including social norm violations)^46^ and that harmful caregiving environments increase the likelihood of delinquent or aggressive behaviors later in life^47,48^. Notably, research examining the relationship between childhood maltreatment and social decision-making in adulthood has predominantly relied on broad constructs such as “antisocial” or “prosocial” behaviors typically assessed via questionnaires, and have rarely examined more specific social decision-making behaviors such as cooperation or punishment.

#### ***How empathy and mentalization shape social decision-making***

Empathy and mentalization play key roles in social interactions, facilitating the exchange of affects and intentions and contributing to the attribution of value to different behavioral choices and their outcomes^2^. Empathy contributes to social decision-making by allowing the sharing of affective information, enhancing attunement to others’ states^49^. According to De Vignemont and Singer^50^, emotional states carry motivational signals that, when detected, improve one’s accuracy in predicting others’ behavior. While not the sole driver of cooperation, empathy serves as a key motivator of prosocial actions^50^. Mentalization enhances the ability to interpret social cues by making the other’s mind accessible^51^. To make appropriate social decisions, we need to consider both how we perceive others and how we believe others perceive us (e.g., “Do I trust them?” or “Will they reciprocate if I choose to share?”)^2^. By generating predictions and expectations about others’ intentions, mentalization improves one’s ability to respond appropriately in social situations^52,53^.

Emerging evidence suggests that empathy and mentalization may help explain how early adversity shapes later social functioning. For instance, Yu et al.^54^ found that empathy and gratitude mediated the association between childhood maltreatment and diminished prosocial behavior in adolescents. Similarly, Masui^55^ showed that childhood adversity was a critical condition under which higher Dark Triad traits—such as psychopathy, characterised by severe empathic deficits—predicted higher levels of online antisocial behavior. Taken together, these findings suggest that disruptions in empathic functioning may be one of the pathways through which childhood maltreatment leads to maladaptive social behaviors.

Alterations in mentalization has also been identified as a mechanism through which childhood adversity may lead to difficulties in social functioning later in life. For instance, Schwarzer et al.^56^ found that lower mentalization skills partially mediated the link between emotional abuse in childhood and aggressive behavior in adulthood. Additional experimental work has shown that individuals with maltreatment histories are more likely to perceive others as threatening and less trustworthy in both facial perception tasks and economic decision-making paradigms, and to show reduced updating of their expectations even when presented with positive feedback (i.e., when interacting with trustworthy partners) in trust-based tasks^57^.

Together, these findings suggest that maltreatment may shape socio-cognitive capacities like empathy and mentalization that guide everyday social decisions. Given the significant number of adults who were raised in contexts marked by neglect or abuse, it is essential to investigate the longer-term consequences of these early social experiences on adult social decision-making and its underlying mechanisms.

### The present study

The present study aims to explore whether disruptions in empathy and mentalization can help explain how early maltreatment severity relates to social decision-making in an adult community sample. Specifically, we investigated whether the severity of self-reported childhood maltreatment is associated with distinct patterns—namely cooperation and punishment—as well as with specific types of social decision-making (e.g., antisocial punishment) in one-shot economic games designed to reflect real-life social dilemmas. We also explored the associations between childhood maltreatment, empathy, and mentalization, and assessed whether empathy and mentalization mediate the link between maltreatment severity and social decision-making. Our hypotheses are outlined as follows:

#### ***Childhood maltreatment severity and social decision-making***

**Fig. 1:**
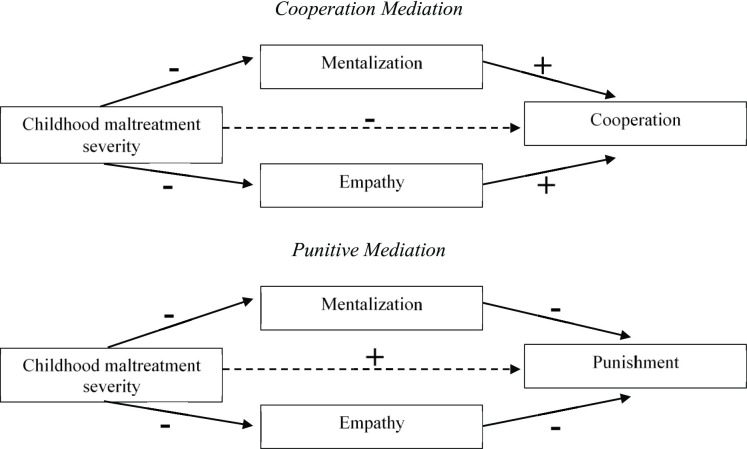
Overview of the proposed mediation models.

We expected maltreatment severity to be negatively associated with the cooperative phenotype (H1a) and across social decision-making tasks related to cooperation (H1b). We predicted that the link between childhood maltreatment severity and cooperative social decision-making would be mediated by mentalization (motivation to mentalize [M-M], self-related mentalization [M-S], and other-related mentalization [M-O]) and empathy (cognitive, resonance, and dissonance). Specifically, we expected negative indirect effects of maltreatment severity on cooperation through lower mentalization and empathy (H2; Fig. 1).

We expected maltreatment severity to be positively associated with the punitive phenotype (H3a) and across social decision-making tasks related to punishment including both norm-enforcing punishment and antisocial punishment (H3b). We predicted that the link between childhood maltreatment severity and punitive social decision-making would be mediated by mentalization (M-M, M-S, M-O) and empathy (cognitive, resonance, and dissonance). Specifically, we expected positive indirect effects of maltreatment severity on punishment through lower mentalization and empathy (H4; Fig. 1).

## Methods

### Participants

Participants were recruited through Amazon Mechanical Turk (MTurk). To be included, participants had to be between 18 and 65 years of age and fluent in English. The survey was hosted on LimeSurvey and completed remotely on participants’ personal devices. A total of 482 individuals initially participated. Following exclusion criteria—namely failing one or both comprehension questions (see socio-demographic questionnaire), completing one or fewer questionnaires or duplicate MTurk IDs (keeping the first entry)—the final sample comprised 327 participants: 174 females (53%) and 153 males (47%). The mean age of participants was 36.4 years (SD = 9.8). Other sociodemographic information can be found in Table 1.

**Table 1:** *Sociodemographic Information*.

Measure		All participants
*N*		327
*Age (M; SD)*		36.4; 9.8
*Sex assigned at birth*	*Male*	153
	*Female*	174
*Ethnicity*	*White*	248
	*African American*	35
	*Other*	44
*Education*	*High school or less*	57
	*College/ Professional Degree*	35
	*University degree*	235
*Employment*	*Employed*	286
	*Unemployed*	41
*Household Income*	*Less than 15 000 USD*	30
	*15 001–30 000 USD*	42
	*30 001–45 000 USD*	62
	*45 001–60 000 USD*	50
	*60 001–75 000 USD*	56
	*More than 75 000 USD*	87

*Note.* Values in the table represent number of participants.

Participants had to read an information and consent form and give their informed consent to proceed. They were informed that compensation would be partially performance-based: in addition to the $1.00 USD base payment, they could earn up to $4.00 from the social decision-making tasks. Upon completion of the study, participants received a unique code to enter on the MTurk platform to receive payment. This study was approved by the Comité d’éthique de la recherche en psychologie et psychoéducation (CÉRPPÉ) of the Université du Québec à Trois-Rivières (reference number: CER-19-258-07), which operates in accordance with the Declaration of Helsinki and is compliant with the requirements of the *Tri-Council Policy Statement: Ethical Conduct for Research Involving Humans (TCPS 2)*. All participants provided informed consent prior to participation.

### Instruments

#### ***Childhood trauma Questionnaire – Short form (CTQ-28)***

The CTQ^58^ is a widely used self-report instrument assessing the severity of five types of childhood maltreatment: emotional, physical, and sexual abuse, as well as emotional and physical neglect. Participants respond on a five-point Likert scale, and each of the five subscales consists of five items (score range: 5–25). A total score is calculated by summing all subscale items (25 items total), with higher scores reflecting more severe childhood maltreatment (range: 25–125). For the purposes of the current study, the total CTQ score was used as a global index of maltreatment severity. This approach aligns with prior research highlighting its adequacy to capture a broad dimension of childhood maltreatment^59^. Internal consistency for the global score was excellent (Cronbach’s α and McDonald’s ω = 0.96). Three additional items, excluded from the global score, are included in the questionnaire to assess whether participants are minimizing their experience. None of our respondents showed a significant score on any of these questions.

#### ***Mentalization Scale (MentS)***

The MentS^29^ is a 28-item self-report measure of mentalization capacity developed by Dimitrijević et al.^29^. It includes three subscales: Motivation to Mentalize (M-M, 10 items), Self-related Mentalization (M-S, 8 items), and Other-related Mentalization (M-O, 10 items). Participants respond using a five-point Likert scale with higher scores reflecting greater mentalizing ability (M-S, M-O) or higher motivation to mentalize (M-M). In the current study, internal consistency was acceptable to excellent: M-M (α = 0.71; ω = 0.62), M-S (α and ω = 0.88), and M-O (α and ω = 0.83). Analyses focused on the three subscales, as they capture distinct components of the construct.

#### ***Affective and cognitive measure of empathy (ACME)***

The ACME^16^ is a 36-item self-report questionnaire designed to measure three components of dispositional empathy: Cognitive Empathy, Affective Resonance, and Affective Dissonance^16^. Participants rate their agreement on a five-point Likert scale. Each subscale includes 12 items (score range: 12–60), with higher scores reflecting greater expression of each component. Cognitive Empathy refers to the capacity to identify and understand others’ emotions. Affective Resonance captures emotional synchrony and physiological congruence with others’ emotional states. Affective Dissonance reflects incongruent emotional reactions (e.g., pleasure in response to others’ distress). Importantly, for Affective Dissonance, higher scores paradoxically reflect *lower* dissonance (i.e., greater empathy), and lower scores suggest greater dissonance. In the present study, internal consistency was satisfactory for all subscales: cognitive empathy (α = 0.85; ω = 0.83), resonance (α and ω = 0.89), and dissonance (α and ω = 0.97).

#### ***Social decision-making tasks***

This study used a series of economic games to assess participants’ cooperative and punitive decisions in one-shot “social dilemma” contexts, following the methodology proposed by Peysakhovich et al.^41^. One-shot iterations allow for the evaluation of individuals’ baseline tendencies—uninfluenced by repeated interactions or reputational concerns—and are considered reflective of stable social heuristics^60^.

Participants completed seven interactive games, each designed to elicit decisions (twelve in total) involving either cooperation or punishment. Decisions were categorized as either cooperative (e.g., sharing, reciprocating, abstaining from harming others) or punitive (e.g., rejecting unfair offers, punishing norm violators).

In each game, participants were assigned a role (e.g., Proposer or Receiver) and asked to allocate points according to game-specific rules. Some games were punishment-free (P-free), allowing « pure » cooperation (e.g., Trust Game, Dictator Game), while others allowed participants to punish others for unfair or even cooperative behavior (e.g., Ultimatum Game, Second-Party Punishment). Table 2 summarizes the structure, if decisions were cooperative or punitive, and motivations associated with each game and decision. *Note: The full wording of game instructions and comprehension questions is available in the Supplementary Materials.*

**Table 2:** *Information about the decision tasks*.

Game	Decision acronym	Structure	Cooperation or punishment
Trust game (P-free)	TG1	Proposer decides to transfer (or not) all their points to a Receiver, trusting they will reciprocate.	Cooperation
	TG2	Points transferred are tripled, and Receiver can share (or not) points with Proposer.	Cooperation
Public Goods Game (P-free)	PGG	Four players decide how many points to contribute to a group project (contributions are doubled and equally divided).	Cooperation
Dictator Game (P-free)	DG	Proposer starts with 100 points and decides how many to give to a Receiver with 0 points (no penalties for not sharing).	Cooperation
Ultimatum Game	UG-C	Proposer offers a share of their 100 points to a Receiver with 0 points.	Cooperation
	UG-MAO	Receiver decides the minimal acceptable offer (MAO) they are willing to accept, (rejecting means 0 points for both players).	Punishment
Second Party Punishment Game	2PP-C	First phase: both players choose to transfer (or not) a sum of points to the other.	Cooperation
	2PP	Second phase: they can punish a non-cooperative player.	Punishment
	2PP-ASP*	Second phase: they can also punish a cooperative player.	Punishment
Third Party Punishment Game	3PP-C*	Proposer chooses whether to take points from a second player. Cooperating means restraining from taking.	Cooperation
	3PP	A third-party observer can punish the proposer for taking (non-cooperating).	Punishment
*All Pay Auction Game*	*AP*	*Players decide how much to invest in a prize (highest spender wins but loses their investment).*	*Competition*

Note. TG: Trust Game; PGG: Public Goods Game; DG: Dictator Game; UG-C: Ultimatum Game-Cooperation; UG-MAO: Minimum accepted offer in the UG; 2PP-C: Second Party Punishment Game-Cooperation; 2PP: Second Party Punishment Game; 2PP-ASP: Second Party Punishment Game-Antisocial Punishment; 3PP-C: Third Party Punishment Game-Cooperation; 3PP; Third Party Punishment Game; AP: All Pay Auction Game (*AP is italicized as it reflects a competitive motivation and, although administered, was not included in the final analyse*). Decisions marked with * refer to 2PP-ASP (Antisocial Punishment) and 3PP-C (cooperating by restraining from taking other’s resources), which were most relevant in our analyses.

After reading the instructions for each game, participants were required to answer a comprehension question to ensure they understood the task. Responses for any game in which the comprehension question was failed were excluded from analysis and treated as missing data. Other responses were retained, provided comprehension was adequate. Overall, 62% of participants passed at least 6 out of 7 comprehension questions.

Each participant completed decisions in all possible roles across the seven games. Decisions were made independently and anonymously. Final compensation was based on a randomly selected decision, with each point valued at $0.025 USD. Specifically, participants were informed that one of the twelve decisions from the social decision-making tasks would be randomly selected to determine their final payment. This incentive method has been shown to enhance engagement in economic game paradigms, independently of the absolute monetary amount involved^61^.

#### ***Socio-demographic questionnaire***

Participants provided general descriptive information, including age, sex assigned at birth, nationality, highest level of education completed, employment status, and household income. Two attention-check questions were presented at the end of the survey to verify data quality. The first required participants to read the entire item and select a specific response; failure to comply led to exclusion from the final sample. The second asked for a numerical answer; participants responding with unrelated words (e.g., “yes” or “no”) were also excluded. It should be noted that a subset of included participants (*n* = 65) did not receive these two items, as these attention checks were introduced later.

### Procedure

To reduce order effects, two separate randomization procedures were implemented. First, the social decision-making tasks were randomized within their block. Second, the three self-report questionnaires (CTQ, MentS, and ACME) were randomized within a separate block. The full study was launched in two waves: in the first, participants completed the economic games before the questionnaires; in the second, the order was reversed. The sociodemographic questionnaire was always completed at the end. Completion time ranged between 35 and 45 minutes.

### Analytic plan

Decisions from social decision-making games in which participants failed the comprehension question were re-coded as missing (see Supplementary Table 2 for details about missing data). No imputation was applied. These decisions were then standardized using z-scores to ensure comparability across tasks with differing point scales. All analyses were conducted using SPSS version 29.0.2.0^62^.

#### ***Factor analysis to identify cooperative and punitive phenotypes***

We conducted an exploratory factor analysis (EFA) with principal axis factoring using varimax rotation on the standardized data to replicate the two-factor structure proposed by Peysakhovich et al.^41^—a cooperation factor and a punitive factor. As in the original study, included decisions were: cooperative decisions without punishment (TG1, TG2, DG, PGG) and norm-enforcing punishment decisions (3PP, 2PP, UG-MAO). However, we did not include the decision associated with competitiveness (AP) as it was shown to be very weakly related to either factor in Peysakhovich et al.^41^; see also Supplementary Fig. 1 for justification). To ensure that the factor structure reflected decisions made with a clear understanding of the task, we temporarily filtered the dataset to include only participants who scored 7/7 on these questions. The EFA was thus conducted on a subsample of participants with full comprehension of the task (*n* = 138).

#### ***Preliminary analyses: identifying mediations paths and covariates***

Preliminary analyses were performed to: (1) preselect which mediation models to run by testing relations between our main study variables (maltreatment severity, mentalization, empathy, social decision-making), and (2) identify potential covariates among our demographic characteristics (sex assigned at birth, age, education level, household income) which would show significant association with these variables. Correlations results were corrected using Benjamini-Hochberg procedure^63^ done within each family of analysis. Specifically, families for the preselection of the mediation models were: (a) correlations between the CTQ and social decision-making decisions (DV-IV paths), (b) correlations between the CTQ and the MentS and ACME measures (DV-mediators paths), and (c) correlations between the MentS, the ACME and social decision-making decisions (mediators-DV paths). For the identification of potential covariates families: (a) correlations (or ANOVAs) with the CTQ, (b) correlations (or ANOVAs) with the MentS and ACME measures and (c) correlations (or ANOVAs) with social decision-making decisions. Significant covariates were retained in selected mediation models.

#### *Mediation analyses*

Mediation models were conducted using PROCESS macro version 5^64^. The goal was to test whether dimensions of mentalization and empathy mediated the association between childhood maltreatment severity (CTQ-total) and social decision-making outcomes: cooperation and punishment factors, as well as individual decision scores when relevant. To reduce the number of tested models, we selected only those supported by our preliminary correlations analysis. In particular, significant associations (*p* < .05, FDR corrected) were required for all three paths of a mediation model: (a) between CTQ-total scores and social decision-making outcomes (IV–DV path), (b) between CTQ-total and the ACME or MentS measures (IV–mediators path), and (c) between the ACME or MentS measures and the social decision-making outcomes (mediators–DV path).

We first tested each mediating variable individually to explore its unique contribution. Then, to test our hypotheses, parallel mediation models were conducted using ordinary least squares (OLS) regressions, allowing simultaneous inclusion of all relevant variables to examine their relative importance.

## Results

### Exploratory factor analysis

**Fig. 2:**
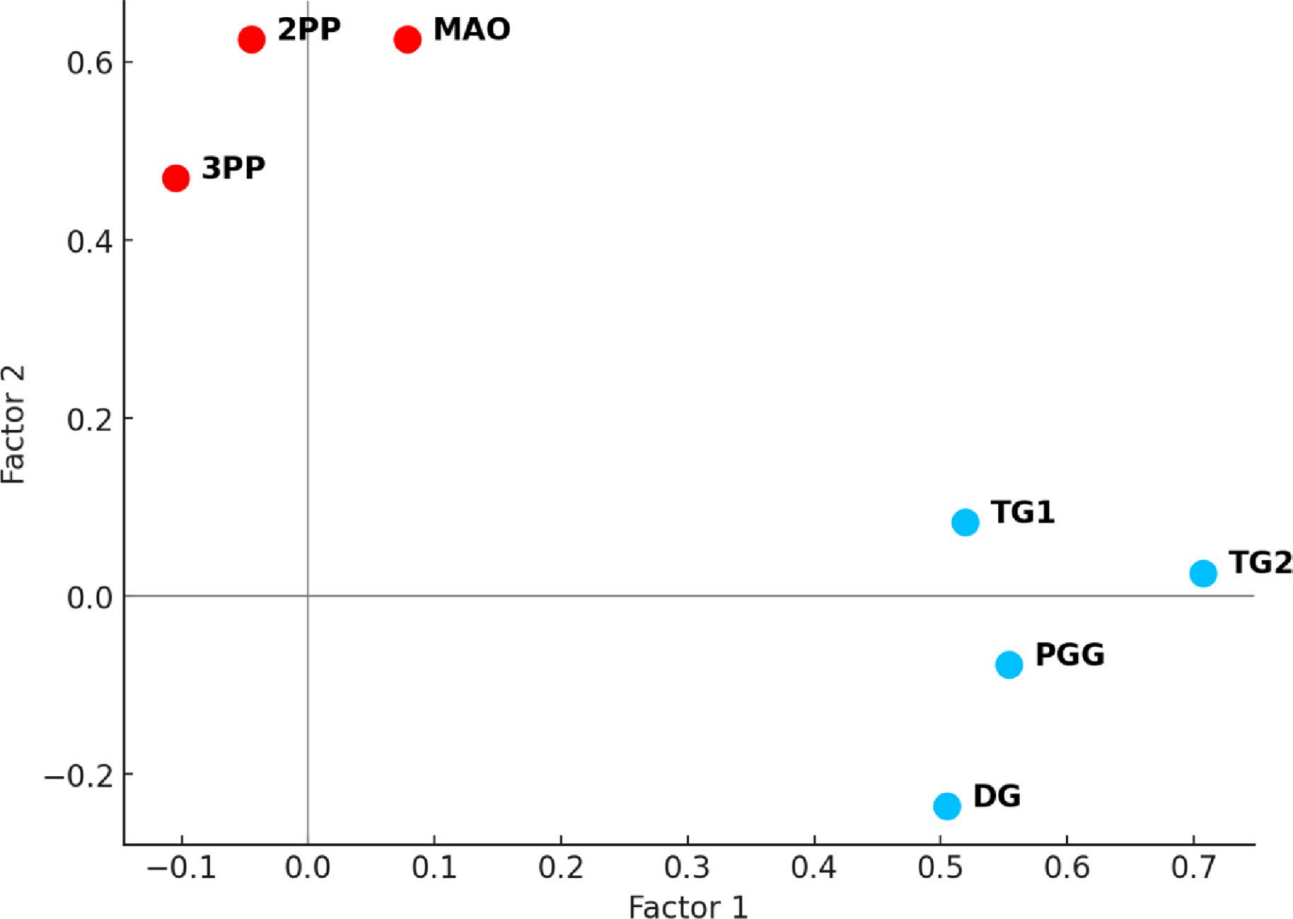
Exploratory Factor Analysis Loadings. *Note*: 3PP: Third Party Punishment Game; 2PP: Second Party Punishment Game; MAO: Minimum accepted offer in the Ultimatum Game; TG: Trust Game; DG: Dictator Game; PGG: Public Goods Game. For more information see Table 2.

All included decisions were sufficiently intercorrelated to warrant EFA (see correlation matrix, Supplementary Fig. 1). As expected, in line with Peysakhovich et al.^41^, a two-factor solution emerged (Fig. 2). The first factor, *Cooperation*, comprised DG, PGG, TG1, and TG2 decisions and accounted for 19.3% of the variance. The second factor, *Prosocial punishment*, comprised UG-MAO, 2PP, and 3PP decisions and explained an additional 15.3% of the variance. Combined, both factors accounted for 34.6% of the total variance. The scree plot revealed a clear leveling off after two factors. Sampling adequacy was acceptable (KMO = 0.65), and Bartlett’s test of sphericity was significant (*p* < .001) indicating that the correlation matrix was suitable for factor analysis.

To compute a factor score for *Cooperation*, participants were required to have answered correctly at least two out of three comprehension questions (one per game, with TG1 and TG2 sharing a single comprehension question). The *Cooperation* factor score was then computed as the mean of standardized responses on TG1, TG2, PGG, and DG. The same procedure was applied for the *Prosocial Punishment* factor. Participants who had correctly answered at least two out of three comprehension questions (corresponding to UG-MAO, 2PP, and 3PP) were included. Their score on the *Prosocial Punishment* factor was computed as the mean of standardized decisions across these three games. These scores were used as factor scores in subsequent analyses.

### Preliminary analyses

Descriptive statistics for child maltreatment severity, mentalization, empathy and social decision-making are presented in Supplementary Tables 1 and in Supplementary Fig. 2.

Correlations between our variables are presented in Table 3. For the IV-DV paths, no significant association was found between CTQ and the *Cooperation* and *Prosocial Punishment* factor scores. Childhood maltreatment severity showed significant associations with three individual decisions: a negative correlation with the *Third-Party Cooperation* decision (3PP-C; *r* = − .33, *p* < .001), a positive correlation with the *Antisocial Punishment* decision (2PP-ASP; *r* = .32, *p* < .001), and a positive correlation with the Public Goods Game (PGG; *r* = 22, *p* = .002). For the IV-mediators paths, CTQ total score was negatively associated with other-related mentalization (*r* = − .15, *p* = .009), self-related mentalization (*r* = − .50, *p* < .001), motivation to mentalize (*r* = − .32, *p* < .001), cognitive empathy (*r* = − .41, *p* < .001), affective resonance (*r* = − .62, *p* < .001), and affective dissonance (*r* = − .62, *p* < .001). For the mediators-DV paths, self-related mentalization (*r* = .43, *p* < .001), motivation to mentalize, (*r* = .26, *p* < .001), cognitive empathy (*r* = .34, *p* < .001), affective resonance (*r* = .54, *p* < .001), and affective dissonance (*r* = .54, *p* < .001) were all positively associated with the *Third-Party Cooperation* decision (3PP-C). Self-related mentalization (*r* = − .35, *p* < .001), cognitive empathy (*r* = − .26, *p* < .001), affective resonance (*r* = − .41, *p* < .001), and affective dissonance (*r* = − .42, *p* < .001) were significantly negatively associated with the *Antisocial Punishment* decision. Cognitive empathy and affective dissonance were significantly negatively associated with the *Ultimatum Game-Cooperation* (UG-C) decision.

**Table 3:** Spearman correlations between main Variables.

	1. Childhood maltreatment severity	2. Other-related Mentalization	3. Self-related Mentalization	4. Motivation to Mentalize	5. Cognitive Empathy	6. Affective Resonance	7. Affective Dissonance	
1	--							
2	− 0.145**	--						
3	− 0.504***	.237^a^	--					
4	− 0.315***	0.613 ^a^	0.401 ^a^	--				
5	− 0.405***	0.598 ^a^	0.543 ^a^	0.571 ^a^	--			
6	− 0.624***	0.380 ^a^	0.683 ^a^	0.587 ^a^	0.644 ^a^	--		
7	− 0.615***	0.201 ^a^	0.687 ^a^	0.443 ^a^	0.514 ^a^	0.830 ^a^	--	
F 1: Coop.	0.127	− 0.052	− 0.082	− 0.081	− 0.141	− 0.011	− 0.070	
F 2: Punish.	− 0.004	0.081	− 0.040	− 0.012	− 0.014	− 0.093	− 0.123	
TG1	0.069	− 0.082	− 0.016	− 0.132	− 0.070	− 0.052	− 0.061	
TG2	0.021	− 0.021	− 0.041	− 0.039	− 0.037	0.062	− 0.015	
PGG	0.218**	− 0.083	− 0.120	− 0.112	− 0.174	− 0.026	− 0.017	
DG	0.092	0.032	− 0.068	0.004	− 0.089	0.006	− 0.076	
UG-C	− 0.079	0.025	0.098	0.043	0.102	0.207*	0.219**	
UG-MAO	− 0.019	0.011	0.037	− 0.021	0.043	− 0.005	− 0.013	
3PP-C	− 0.332***	0.104	0.430***	0.260***	0.336***	0.536***	0.538***	
3PP	0.099	0.151	− 0.134	0.012	− 0.094	− 0.164	− 0.177	
2PP-C	0.063	0.046	− 0.009	− 0.008	0.025	0.006	− 0.045	
2PP	0.066	− 0.063	− 0.109	− 0.116	− 0.145	− 0.165	− 0.193*	
2PP-ASP	0.319***	0.059	− 0.350***	− 0.140	− 0.257***	− 0.406***	− 0.420***	
AP	0.034	0.111	− 0.019	0.017	− 0.021	− 0.023	− 0.110	

Note. 3PP: Third Party Punishment Game; 3PP-C: Third Party Punishment Game-Cooperation; 2PP: Second Party Punishment Game; 2PP-C: Second Party Punishment Game-Cooperation 2PP-ASP: Second Party Punishment Game-Antisocial Punishment; MAO: Minimum accepted offer in the Ultimatum Game; TG: Trust Game; DG: Dictator Game; PGG: Public Goods Game; AP: All Pay Auction Game; F 1-Coop.: Factor 1 Cooperation; F 2-Punish.: Factor 2 Punishment. *p < .05. **p < .01. ***p < .001 FDR (Benjamini-Hochberg) adjusted. ^a^ Indicate significant correlations but not corrected because not part of our analysis plan.

Based on these preliminary results we conducted mediation analyses assessing: (a) CTQ scores association with the *Third-Party Cooperation* decision with self-related mentalization, motivation to mentalize and all three dimension of dispositional empathy as mediators; and (b) CTQ scores association with the *Antisocial Punishment* decision with self-related mentalization and all three dimension of dispositional empathy as mediators. Results for preliminary analyses regarding covariates can be found in Supplementary Tables 3 and 4. Included covariates for each tested mediation model can be found in Supplementary Table 5.

### Mediation analyses

#### ***Third-Party Cooperation mediation model***

**Simple mediations.** Five exploratory simple mediation models were tested to assess whether specific empathy- and mentalization-related processes mediated the association between childhood maltreatment severity and cooperation in the 3PP-C task. All variables—motivation to mentalize, self-related mentalization, cognitive empathy, affective resonance, and affective dissonance—were significant mediators when tested individually. Affective empathy components (resonance and dissonance) fully mediated the relationship, while motivation to mentalize, self-related mentalization and cognitive empathy emerged as partial mediators. Higher maltreatment severity predicted lower scores on each construct, which in turn were associated with reduced cooperation. Full statistical details and figures are available in the Supplementary Fig. 3.

**Fig. 3:**
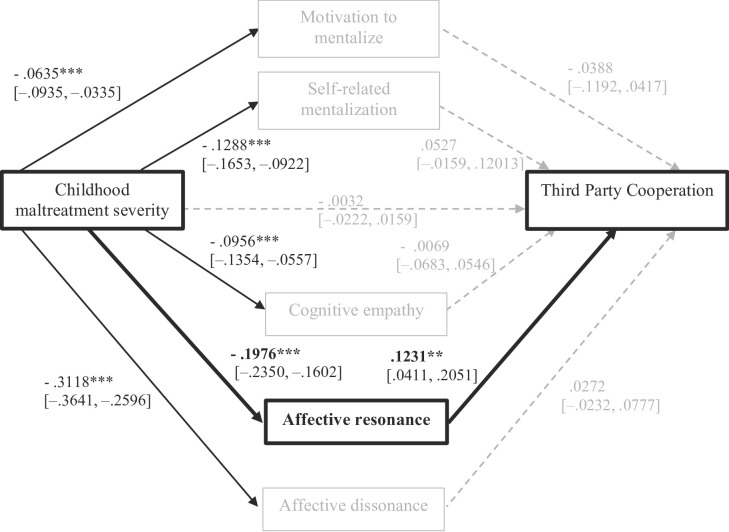
Parallel mediation model for Third Party Cooperation decision (3PP-C). *Note*: Indirect effects all nonsignificant, except for: TOTAL = − 0.0365, 95% CI [- 0.0577, − 0.0226] and Affective resonance = − 0.0243, 95% CI [- 0.0471, − 0.0097]. Education, Income, Sex, Age and Employment as covariables. * *p* < .05; ** *p* < .01; *** *p* < .001. Grey dashed lines represent relations that are not significant. *N* = 257.

**Parallel mediation.** To test our main hypotheses, we entered all five mediators simultaneously (Fig. 3). When all five mediators were entered simultaneously, the total indirect effect of childhood maltreatment severity on *Third-Party Cooperation* (3PP-C) remained significant (b = –0.0365, 95% CI [–0.0577, –0.0226]), indicating that the mediators collectively explained part of the association. However, only affective resonance emerged as a unique and significant mediator in the combined model (b = –0.0243, 95% CI [–0.0471, –0.0097]), suggesting that this pathway primarily drives the observed mediation effect. The indirect effects via motivation to mentalize, self-related mentalization, cognitive empathy, and affective dissonance were not significant when controlling for shared variance among mediators. The direct effect was nonsignificant (b = –0.0032, *p* = .74) suggesting a full mediation pattern where CTQ predicts lower affective resonance which in turn is associated with lower odds of choosing to cooperate in this task.

#### ***AntisocialPunishment*** ***mediationmodel***

**Simple mediations.** We tested four exploratory simple mediation models to examine whether specific empathy- and mentalization-related processes mediated the relationship between childhood maltreatment severity and *Antisocial Punishment* (2PP-ASP). Significant indirect effects were found for self-related mentalization, affective resonance, and affective dissonance, while the pathway through cognitive empathy was not significant. Self-related motivation and affective resonance showed partial mediation, while affective dissonance fully mediated the association. In all three cases, higher maltreatment severity predicted lower scores on the mediator, which in turn was associated with greater Antisocial Punishment. Full statistical details and visual representations are available in the Supplementary Fig. 4.

**Fig. 4:**
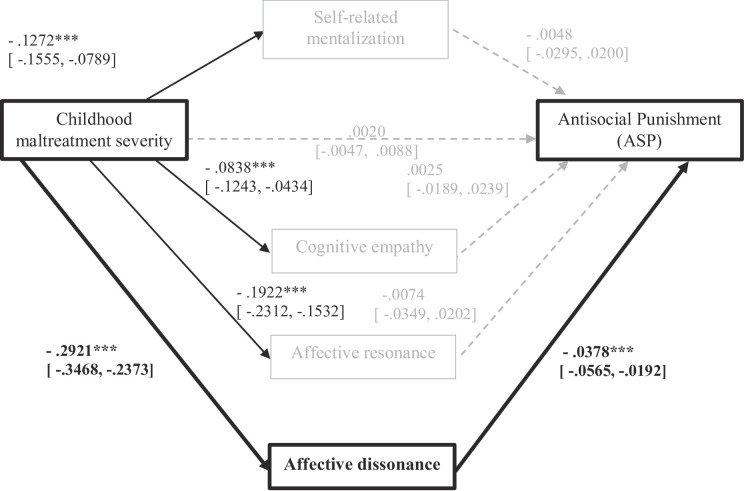
Parallel Mediation Model for Antisocial Punishment decision (2PP-ASP). *Note*: Indirect effects all nonsignificant, except for: TOTAL = 0.0128, 95% CI [ 0.0082, 0.0188] and Dissonance = 0.0111, 95% CI [ 0.0052, 0.0182]. Education, Income, Sex, Age and Employment as covariables. * *p* < .05; ** *p* < .01; *** *p* < .001. Grey dashed lines represent relations that are not significant. *N* = 228.

**Parallel mediation.** We then tested our main hypotheses with a parallel mediation model including the four mediators: self-related mentalizing, cognitive empathy, affective resonance, and affective dissonance (Fig. 4). The overall indirect effect was significant (b = 0.0128, 95% CI [0.0082, 0.0188]), but only dissonance remained a significant individual mediator in the combined model (b = 0.0111, 95% CI [0.0052, 0.0182]). The indirect effects of self-related mentalization, affective resonance and cognitive empathy were no longer significant when accounting for shared variance. The direct effect was nonsignificant (b = 0.0020, *p* = .55) suggesting a full mediation pattern where CTQ predicts greater use of *Antisocial Punishment* through its relation with lower affective dissonance scores, reflecting greater affective dissonance (i.e., lower empathy).

## Discussion

This study aimed to better understand how childhood maltreatment shapes social decision-making in adulthood through key underlying psychological processes. More specifically, we examined how the severity of self-reported childhood maltreatment is associated with cooperation and punishment behaviors in a general population of adults, and whether these associations are mediated by empathy and mentalization. In doing so, we sought to explore if and how maltreatment history is related to the way adults navigate social dilemmas—contexts that require interpreting others’ emotions, reflecting on one’s own mental states, and balancing personal interests with social norms. Contrary to our initial hypotheses—which anticipated broad effects of childhood maltreatment on social decision-making—our findings revealed a more selective pattern. Rather than uniformly decreasing cooperation or increasing punitive behaviors, maltreatment severity was specifically linked to two decisions: (1) reduced restraint in taking another player’s resources while being observed by a third-party enforcer (3PP-C, reduced cooperation), and (2) heightened punishment of cooperative partners (2PP-ASP, antisocial punishment). These findings suggest that early adversity may exert its strongest influence in morally salient and socially regulated contexts, rather than broadly impairing all forms of social decision-making. Although childhood maltreatment effects were mediated by empathy and mentalization, when controlling for each other’s influence, the effect was only mediated by affective empathy, which partly contradicted our hypotheses that both empathy and mentalization would mediate these relations. One of the study’s contributions lies in its empirical support for the dual affective empathy model proposed by Vachon and Lynam^16^. Our results indicate that distinct emotional processes—affective resonance and dissonance—may differentially mediate the link between childhood maltreatment and social behaviors.

The Third-Party Punishment (3PP) game provides a structured context to examine how individuals behave when fairness is externally monitored and social norms are enforced^65^. In this task, an active player makes decisions that affect a passive other, while a third-party observer monitors the interaction and holds the authority to punish unfair behavior. The 3PP-C decision specifically reflects whether the active player chooses to cooperate—by refraining from taking the passive player’s resources—or to act selfishly and take them, despite being observed. This setting introduces a strong social deterrent: individuals are not only aware of the potential for punishment but may also anticipate reputational consequences or negative social judgment^66,67^. Notably, taking benefits the active player less than it harms the passive one, adding an additional layer of moral salience that may further discourage self-serving behavior. In our study, higher severity of childhood maltreatment was associated with a lower likelihood of cooperating in this context (i.e., reduced restraint from taking from the passive player). In other words, individuals with higher maltreatment severity were more inclined to seize resources from others when given the opportunity, resulting in an unequal distribution of resources that favors themselves at the expense of others—all the while under social scrutiny and despite clear deterrents. Mediation analysis suggests that this tendency may be related to diminished affective resonance—the tendency to be emotionally attune to others and register their distress. This dimension of affective empathy plays a central role in supporting social adaptation, allowing individuals to anticipate the impact of their behavior on others and adjust accordingly^50^. Although childhood adversity has sometimes been associated with increased affective empathy, particularly when assessed as personal distress (self-oriented affective empathy), other-oriented affective empathy is often found to be reduced in adulthood^26^. The present findings align more closely with this latter pattern, given that other-oriented measures of affective empathy are conceptually and empirically closer to our measures of affective resonance (see correlation reported in^16^).

These findings suggest that childhood maltreatment may be associated to a disrupted development of affective capacities that support norm adherence. A diminished ability or tendency to “read the room”—to intuitively register the emotional implications of one’s actions—may increase the risk of socially inappropriate or self-centered decisions. Interestingly, these findings echo theoretical frameworks that conceptualize norm-deviant behavior as motivated, at least in part, by a desire to maximize self-interest. One such framework is the Dark Factor of Personality (D), proposed by Moshagen et al.^68^, which refers to a general tendency to pursue personal gain even when doing so harms others—often justified by self-serving beliefs. In our study, participants exposed to more severe childhood maltreatment were more likely to make self-benefiting choices that violated fairness norms. Rather than attributing these behaviors solely to fixed personality traits, our results suggest a developmental pathway rooted in early relational adversity. Specifically, reduced affective resonance—associated with higher maltreatment severity—may limit their ability to perceive or attend to the emotional impact of their actions, thereby fostering a greater tolerance for deceiving others when personal gains are at stake. In this view, the seeds of norm-violating behavior may lie not in a dispositional disregard for others, but in developmental disruptions in emotional and interpersonal functioning. This trauma-sensitive interpretation offers an alternative lens through which to view “dark” behaviors. Rather than reflecting inherent characteristics (e.g., callousness or moral disengagement), they may reflect maladaptive adaptations to unpredictable or threatening early environments—adaptations that prioritize self-preservation over social attunement. Given the cross-sectional nature of our findings, longitudinal studies incorporating personality measure are needed to test this hypothesis.

The Second Party Punishment Game offers a framework to examine responses to reciprocal behavior in a dyadic exchange. In the first phase, both participants simultaneously decide whether to transfer a portion of their points to the other player, creating the potential for cooperation. In the second phase, participants can choose to punish their partner—at a cost to themselves—based on the partner’s decision in the initial exchange. In the standard 2PP decision, punishment is directed only toward noncooperative partners, reflecting a response to perceived unfairness or norm violation. In the extended 2PP-ASP decision, participants also have the option to punish cooperative partners, allowing for the investigation of atypical or maladaptive punishment patterns—antisocial punishment^41^. Unlike noncooperative decisions in *Third-Party Cooperation*—which reflect a disregard for social norms in favor of self-interest—*Antisocial Punishment* is more overtly norm-violating: it involves a direct violation of fairness norms by sanctioning prosocial behavior. This paradoxical behavior raises questions about the underlying psychological processes that drive someone to punish not transgressions, but fairness itself. In our study, greater severity of childhood maltreatment was associated with an increased tendency to engage in antisocial punishment. Mediation analysis revealed that this association was mediated by affective dissonance, or *Schadenfreude*—the experience of pleasure at another person’s distress. This finding suggests that early relational trauma may heighten dissonant affective responses, which then fuel antisocial punitive behavior. The causal nature of this proposed pathway could be formally tested using longitudinal designs.

These findings align with social-functional models of Schadenfreude, which propose that this emotion may serve to regulate perceived social hierarchies^69^—particularly when the other is seen as dominant or morally superior. From this perspective, punishing a cooperative partner may reflect a psychological attempt to reassert control, challenge a perceived imbalance of virtue, or preemptively defend against social threat. This mechanism may be especially prominent in individuals with histories of relational trauma, for whom experiences of fairness may feel unfamiliar, destabilizing, or even threatening. In such cases, Schadenfreude may operate as an internal signal that legitimizes norm-violating behavior, especially when alternative regulatory strategies are underdeveloped or absent. Contrary to traditional accounts that attribute antisocial punishment to a fundamental detachment from moral concern (as seen in personality traits like those from the Dark Triad^70^), rather than suggesting an absence of empathy, our findings align with the dual model of affective empathy proposed by Vachon and Lynam^16^. In this framework, affective dissonance—manifested as pleasure in another’s suffering—represents not a lack of emotional attunement, but a reversal of its typical emotional valence—an emotional response misaligned with social norms, yet deeply rooted in prior interpersonal experience. This interpretation invites a more compassionate and developmentally grounded perspective—one that recognizes how atypical expressions of empathy may emerge in the wake of disrupted caregiving experiences, and how they may underlie behaviors typically viewed as morally deviant.

Our results replicated the phenotypic structure proposed by Peysakhovich et al.^41^, which posits that individuals exhibit consistent behavioral tendencies across social dilemmas—namely, a general propensity to cooperate or to punish. From our factor analysis, we show that in our sample, those who cooperated or punished in one task tended to do so in others, reinforcing the structural coherence of the model. However, these phenotypic components showed little association with study variables (childhood maltreatment, mentalization, and empathy). These findings suggest that while Peysakhovich et al.’s model^41^ captured general patterns of social behavior, it may be less sensitive to motivational, affective, or developmental factors—particularly those linked to childhood early adversity.

### Limitations

While this study offers methodological strengths worth highlighting, a few limitations warrant consideration. First, while cross-sectional in design, it serves as a valuable starting point for identifying plausible psychological pathways linking childhood maltreatment to adult social decision-making. Such designs are particularly well-suited for exploratory models and can generate testable hypotheses for future longitudinal research^71^. Second, the use of self-report questionnaires and online data collection enabled us to reach a broad and diverse nonclinical sample, and allowed for the investigation of latent psychological processes (e.g., empathy dissonance, mentalization) that are often difficult to access through behavioral tasks alone^72^. That said, this approach limits the possibility of clinical verification (e.g., psychiatric diagnoses, implicit biases in mentalization), and future studies could benefit from incorporating multi-method or clinical assessments. An important strength of our study, however, lies in the inclusion of social decision-making tasks inspired by the economic game design of Peysakhovich et al.^41^, which provide ecologically valid behavioral measures and help diversify our methodological approach. Third, our sample reported relatively high levels of childhood maltreatment (M = 53.12) and lower-than-average scores in empathy (Cognitive empathy: M = 43.66; Affective Resonance: M = 47.04; Affective Dissonance: M = 46.63) and self-related mentalization (M = 25.10) compared to normative data^16,29,73^. This may reflect a recruitment bias (perhaps linked to the study’s title or framing) or it could signal a broader pattern, where individuals with trauma histories may be underrepresented in normative samples. Although this increases strength of trauma-related effects, it may also limit the generalizability of our findings. Fourth, our study did not differentiate between specific types of maltreatment or their timing. Prior work has shown that the nature, severity, and timing of adverse experiences can have differential impacts on socio-affective outcomes (e.g., Russotti et al.^74^). While this level of detail was beyond the scope of the present study, future research would benefit from finer-grained assessments of maltreatment characteristics. Also, we used self-report questionnaires to assess empathy and mentalization. These measures capture dispositional or trait dimensions of these constructs reflecting tendencies or perceived abilities rather than actual performance. Incorporating behavioral tasks would provide a more direct assessment of empathy and mentalization in action, and could help clarify if and how childhood maltreatment affects participant’s capacity to empathize and mentalize during social interactions and the impact of these disruptions on social-decision making. Fifth, our choice of measures presents some limitations. Childhood maltreatment was assessed retrospectively, which may introduce recall bias for negative memories influenced by current psychological state (e.g., depression^75^). However, prior work indicates that retrospective reports of childhood maltreatment with the CTQ show substantial temporal stability and limited state dependence across clinical and nonclinical populations^76^. Furthermore, while variables such as current or past psychopathology and trauma during adulthood could be related to our mentalization, empathy and social decision-making measures, we did not control for these. Future studies incorporating prospective or multi-informant measures of childhood maltreatment and traumatic events during adulthood in addition to measures of psychopathology would help address this limitation. Finally, future work could look into how other types of mentalization such as trauma-specific mentalization (e.g.,^77,78^) could be involved in the relation between childhood maltreatment and social decision-making.

## Conclusion

Our study contributes to a growing body of work advocating for a developmental and trauma-sensitive lens in understanding adult social behavior. Rather than pathologizing norm-deviant choices in isolation, it calls for a deeper inquiry into the histories that shape them—and the psychological mechanisms that might support their transformation.

Our findings highlight potential specific pathways through which childhood maltreatment could influence adult social behavior—particularly in contexts where prosocial norms are challenged. By identifying distinct affective empathy mechanisms—namely affective resonance and dissonance—this study offers a nuanced understanding of how early adversity may be related to increased antisocial or self-serving behaviors in adulthood.

These insights underscore the importance of considering empathy and mentalization not only as mediators of trauma’s long-term effects, but also as promising intervention targets. Effective interventions may therefore need to engage both the *feeling* and the *thinking* dimensions of social functioning when working with individuals with complex trauma histories—whether through prevention, early intervention, or clinical treatment.

## Supplementary Information

Below is the link to the electronic supplementary material.

Supplementary Material 1

## Data Availability

The datasets used and/or analysed during the current study are available from the corresponding author on reasonable request.
