# Supplementary material for: Empathy and mentalization as mediators between childhood maltreatment and social decision-making during adulthood
Source: Sci Rep. 2026 Feb 14;16:9111. doi: 10.1038/s41598-026-37273-9 (PMC12996610; doi:10.1038/s41598-026-37273-9)
Supplement: Supplementary file 1 — Supplementary Material 1 [file 41598_2026_37273_MOESM1_ESM.docx]

**Supplementary Materials**

#### **Supplementary Figure 1** Correlation between the different decisions

***
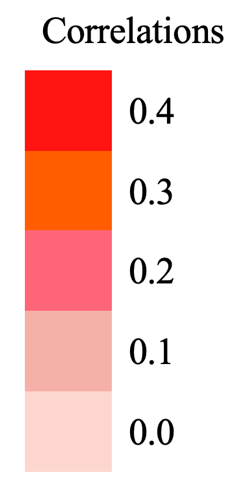
***
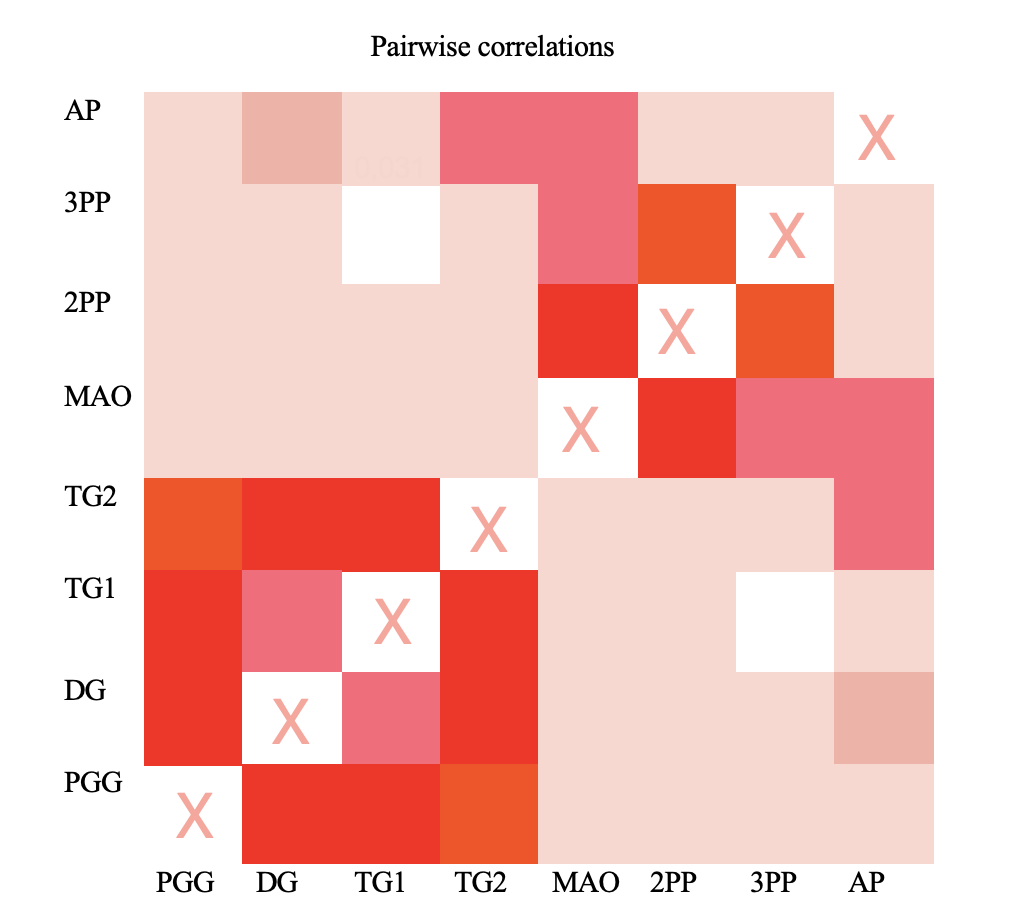


*Notes.* AP : All Pay Auction Game; 3PP: Third Party Punishment Game; 2PP: Second Party Punishment Game; MAO: Minimum accepted offer in the Ultimatum Game; TG: Trust Game; DG: Dictator Game; PGG: Public Goods Game. For more information see Table 2. Although AP was not included in the EFA analysis, its relation to other decisions is presented here. The AP decision was removed from the final FA because while AP provides valuable information about competitive behavior, it targets a construct conceptually distinct from the two dimensions central to the current study—cooperation and punishment. As illustrated in the figure, AP did not load meaningfully on either principal component and therefore was excluded from the final EFA model to preserve the interpretability and coherence of the component structure.

**Supplementary Table 1**

*Descriptive statistics of Main Variables*

|  | N | Min | Max | Mean | SD |
| --- | --- | --- | --- | --- | --- |
| CTQ- Emotional Abuse^1^ | 327 | 5 | 25 | 11.82 | 5.70 |
| CTQ- Physical Abuse^1^ | 327 | 5 | 25 | 10.41 | 5.93 |
| CTQ- Sexual Abuse^1^ | 327 | 5 | 25 | 9.64 | 5.95 |
| CTQ- Emotional Neglect^1^ | 327 | 5 | 25 | 11.22 | 4.69 |
| CTQ- Physical Neglect^1^ | 327 | 5 | 23 | 10.02 | 4.49 |
| **CTQ Total Score** | 327 | **25** | **119** | **53.12** | **21.89** |
| MentS – Others (M-O) | 327 | 22 | 50 | 39.33 | 5.18 |
| MentS – Self (M-S) | 327 | 11 | 40 | 25.10 | 7.06 |
| MentS – Motivation (M-M) | 327 | 15 | 49 | 37.97 | 5.11 |
| MentS – Total Score^1^ | 327 | 63 | 138 | 102.40 | 13.46 |
| ACME Cognitive Empathy | 327 | 23 | 60 | 43.66 | 7.12 |
| ACME Affective Resonance | 327 | 31 | 60 | 47.04 | 8.73 |
| ACME Affective Dissonance | 327 | 18 | 60 | 46.63 | 12.83 |
| F 1 : Coop. | 275 | -1.57 | 1.47 | - .00 | 0.74 |
| F 2 : Punish. | 253 | -1.41 | 1.73 | - .03 | 0.74 |
| TG1 | 271 | 0 | 1 | .62 | .49 |
| TG2 | 271 | 0 | 150 | 64.92 | 35.29 |
| PGG | 198 | 0 | 100 | 32.80 | 31.66 |
| DG | 314 | 0 | 50 | 28.40 | 17.89 |
| UG-C | 243 | 0 | 50 | 36.81 | 14.86 |
| UG-MAO | 243 | 0 | 50 | 26.95 | 15.23 |
| 3PP-C | 257 | 0 | 1 | .63 | .49 |
| 3PP | 257 | 0 | 100 | 39.34 | 31.24 |
| 2PP-C | 228 | 0 | 1 | .79 | .41 |
| 2PP | 228 | 0 | 70 | 31.70 | 26.62 |
| 2PP-ASP | 228 | 0 | 70 | 13.68 | 19.93 |
| AP | 272 | 0 | 100 | 53.91 | 28.87 |

#### Notes. CTQ: Child Trauma Questionnaire; ACME: Affective and Cognitive Measure of Empathy; F1-2: Factors 1 and 2 from the EFA; TG1-2: Trust Game 1-2; PGG: Public Goods Game; DG: Dictator Game; UG-C: Ultimatum Game cooperation; UG-MAO: Minimum accepted offer in the Ultimatum Game; 3PP-C: Third Party Punishment Game-Cooperation; 3PP: Third Party Punishment Game; 2PP:-C Second Party Punishment Game-Cooperation; 2PP-C: Second Party Punishment Game-Cooperation; 2PP: Second Party Punishment Game; 2PP-ASP: Second Party Punishment Game-antisocial punishment; AP: All Pay Auction Game. For more information see Table 2. ^1^Denotes measures that were not used in the analyses and that are only reported for descriptive purposes.

**Supplementary Figure 2** Distributions of CTQ scores

**
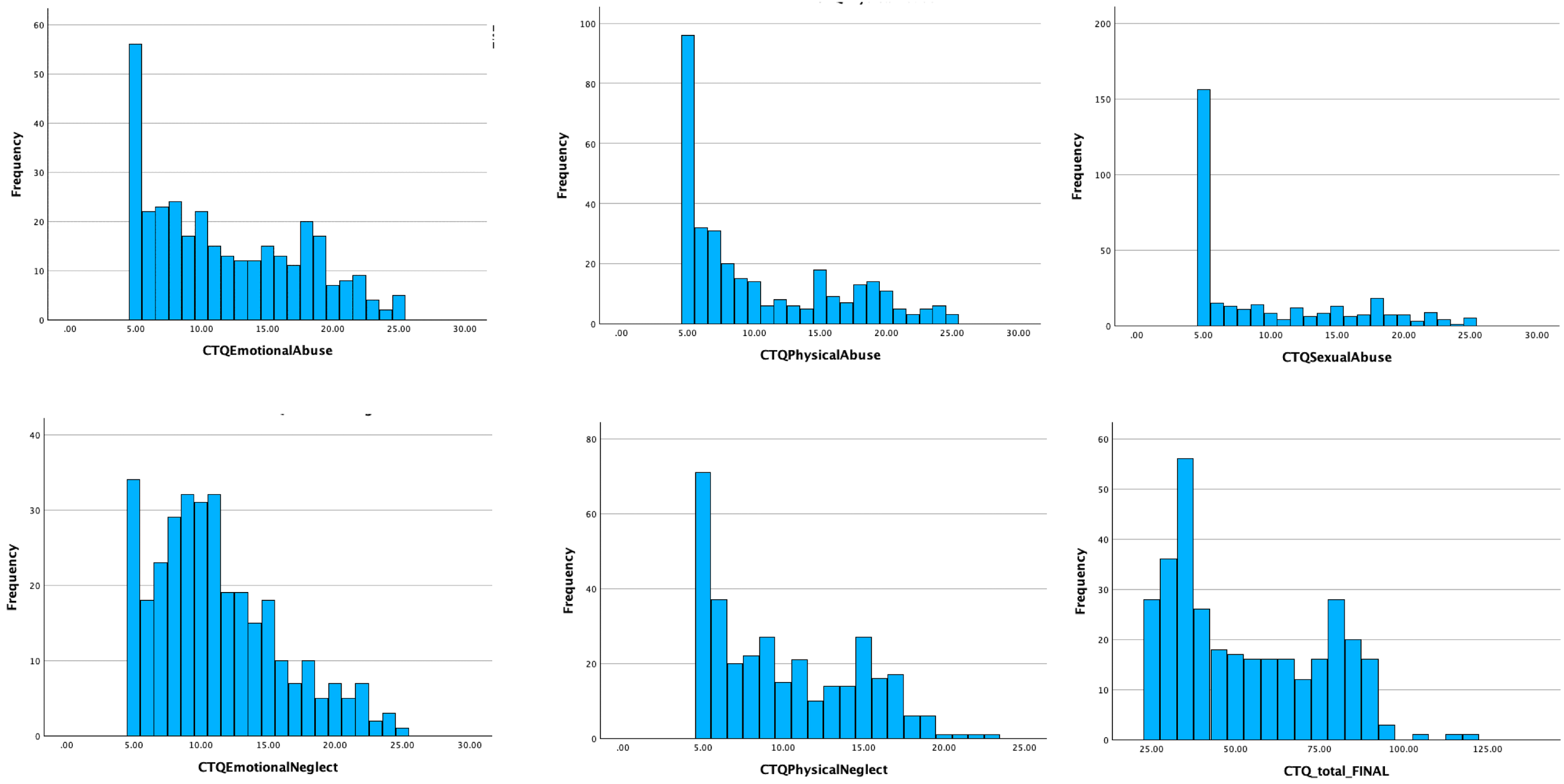
**

**Supplementary Table 2**

Percentage of missing data per decisions

| Decision | % |
| --- | --- |
| TG | 17.3 |
| PGG | 39.8 |
| UG | 26.1 |
| DG | 4.3 |
| 3PP | 21.6 |
| 2PP | 30.1 |
| AP | 17.3 |

*Notes.* TG: Trust Game; PGG: Public Goods Game; UG: Ultimatum Game; DG: Dictator Game; 3PP: Third Party Punishment; 2PP: Second Party Punishment Game; AP: All Pay Auction Game

**Supplementary Table 3**

*Correlation between Main Variables and Socio-Dememographic Variables*

|  | Sex assigned at birth | Age | Education level | Income |
| --- | --- | --- | --- | --- |
| CTQ | -0.036 | -0.103 | .111 | **-.173*** |
| M-O | .026 | -.046 | .033 | .069 |
| M-S | .073 | **.190**** | **-.216***** | **.150*** |
| M-M | **.144^*^** | -.016 | -.110 | .065 |
| COG | **.126*** | .079 | **-.171**** | **.130*** |
| RES | **.190^**^** | .108 | **-.270***** | **.156*** |
| DISS | **.216^***^** | **.151^*^** | **-.346^***^** | .098 |
| F 1: Coop. | -.168 | .113 | .109 | -.013 |
| F 2: Punish. | **-.200*** | -.058 | .169 | .022 |
| TG1 | -.097 | .026 | .043 | .026 |
| TG2 | -.153 | .085 | .090 | -.031 |
| PGG | -.079 | .111 | .134 | .015 |
| DG | -.091 | .094 | .074 | -.086 |
| UG-C | -.119 | .127 | -.006 | .030 |
| UG-MAO | -.094 | .055 | .085 | .088 |
| 3PP-C | .106 | .041 | **-.285***** | .101 |
| 3PP | -.123 | -.109 | .147 | -.062 |
| 2PP-C | -.070 | .098 | .147 | .044 |
| 2PP | **-.135**** | -.045 | .148 | .131 |
| 2PP-ASP | .026 | -.058 | .149 | .031 |
| AP | -.022 | .038 | **.191*** | .054 |

*Note.* CTQ: Child Trauma Questionnaire; M-O: Mentalization-Other; M-S: Mentalization-Self; M-M: Motivation to mentalize; COG: Cognitive empathy; RES: Affective resonance; DIS: affective dissonance; F1-2: Factors 1 and 2 from the EFA; TG1-2: Trust Game 1-2; PGG: Public Goods Game; DG: Dictator Game; UG-C: Ultimatum Game cooperation; UG-MAO: Minimum accepted offer in the Ultimatum Game; 3PP-C: Third Party Punishment Game-Cooperation; 3PP: Third Party Punishment Game; 2PP:-C Second Party Punishment Game-Cooperation; 2PP-C: Second Party Punishment Game-Cooperation; 2PP: Second Party Punishment Game; 2PP-ASP: Second Party Punishment Game-antisocial punishment; AP: All Pay Auction Game. For more information see Table 2.

*p < .05, **p < .01, ***p < .001 FDR (Benjamini-Hochberg) adjusted.

**Supplementary Table 4**

*ANOVA Results for Socio-demographic Variables*

| *Variable* | *F* | *p* | η² |
| --- | --- | --- | --- |
| *Ethnicity and* |  |  |  |
| CTQ | 0.239 | .788 | .001 |
| M-O | 5.558 | .004* | .033 |
| M-S | 0.944 | .390 | .006 |
| M-M | 2.518 | .082 | .015 |
| COG | 3.046 | .049 | .018 |
| RES | 0.504 | .605 | .003 |
| DISS | 1.117 | .329 | .007 |
| F 1: Coop. | 0.215 | .807 | .002 |
| F 2: Punish. | 3.231 | .041 | .025 |
| TG1 | 0.139 | .871 | .001 |
| TG2 | 0.143 | .866 | .001 |
| PGG | 1.867 | .157 | .019 |
| DG | 0.098 | .907 | .001 |
| UG-C | 2.321 | .100 | .019 |
| UG-MAO | 1.206 | .301 | .010 |
| 3PP-C | 2.497 | .084 | .019 |
| 3PP | 4.119 | .017 | .031 |
| 2PP-C | 3.295 | .039 | .028 |
| 2PP | 0.937 | .393 | .008 |
| 2PP-ASP | 3.911 | .021 | .034 |
| AP | 0.251 | .778 | .002 |
| *Employment and* |  |  |  |
| CTQ | 3.239 | .073 | .010 |
| M-O | 1.689 | .195 | .005 |
| M-S | 3.929 | .048 | .012 |
| M-M | 8.125 | .005* | .024 |
| COG | 0.655 | .419 | .002 |
| RES | 25.043 | < .001*** | .072 |
| DISS | 25.678 | < .001*** | .073 |
| F 1: Coop. | 1.292 | .257 | .005 |
| F 2: Punish. | 1.198 | .275 | .005 |
| TG1 | 1.274 | .260 | .005 |
| TG2 | 1.666 | .198 | .006 |
| PGG | 0.031 | .861 | .000 |
| DG | 0.271 | .603 | .001 |
| UG-C | 0.042 | .838 | .000 |
| UG-MAO | 0.792 | .374 | .003 |
| 3PP-C | 13.801 | < .001** | .051 |
| 3PP | 0.219 | .641 | .001 |
| 2PP-C | 0.499 | .481 | .002 |
| 2PP | 1.992 | .159 | .009 |
| 2PP-ASP | 0.536 | .465 | .002 |
| AP | 1.046 | .307 | .004 |

*Notes.* CTQ: Child Trauma Questionnaire; M-O: Mentalization-Other; M-S: Mentalization-Self; M-M: Motivation to mentalize; COG: Cognitive empathy; RES: Affective resonance; DIS: affective dissonance; F1-2: Factors 1 and 2 from the EFA; TG1-2: Trust Game 1-2; PGG: Public Goods Game; DG: Dictator Game; UG-C: Ultimatum Game cooperation; UG-MAO: Minimum accepted offer in the Ultimatum Game; 3PP-C: Third Party Punishment Game-Cooperation; 3PP: Third Party Punishment Game; 2PP:-C Second Party Punishment Game-Cooperation; 2PP-C: Second Party Punishment Game-Cooperation; 2PP: Second Party Punishment Game; 2PP-ASP: Second Party Punishment Game-antisocial punishment; AP: All Pay Auction Game. For more information see Table 2.

*p < .05, **p < .01, ***p < .001 FDR (Benjamini-Hochberg)

**Supplementary Table 5** List of covariates included in each mediation model

| Mediation Models | Included covariates |
| --- | --- |
| **3PP-C: parallel mediation model** | Education level; Income; Sex; Age; Employment |
| 3PP-C: motivation to mentalize model | Education level; Income; Sex; Employment |
| 3PP-C: self-mentalization model | Education level; Income; Age; Employment |
| 3PP-C: cognitive empathy model | Education level; Income; Sex; Employment |
| 3PP-C: affective resonance model | Education level; Income; Sex; Employment |
| 3PP-C: affective dissonance model | Education level; Income; Sex; Age; Employment |
|  |  |
| **2PP-ASP: parallel mediation model** | Education level; Income; Sex; Age; Employment |
| 2PP-ASP: self-mentalization model | Education level; Income; Age |
| 2PP-ASP: cognitive empathy model | Education level; Income; Sex |
| 2PP-ASP: affective resonance model | Education level; Income; Sex; Employment |
| 2PP-ASP: affective dissonance model | Education level; Income; Sex; Age; Employment |

*Note.* Bold indicate models testing our main hypotheses. 3PP-C: Third Party Punishment Game-Cooperation; 2PP-ASP: Second-Party Punishment Game-Antisocial Punishment.

**Supplementary Figure 3** Results from the Simple Mediation Analyses on 3PP-C

Childhood maltreatment severity

Third Party Cooperation

- .0630***

[- .0930, - .0330]

Motivation to Mentalize

.0646***

[.0045, .1247]

- .0321***

[- .0468, - .0174]

Indirect effect = - .0041, 95% CI [- .0094, .0000].

Education, Income, Employment and Sex as covariables.

Childhood maltreatment severity

Third Party Cooperation

Self-related Mentalization

.1216***

[.0679, .1753]

- .1288***

[- .1652, - .0923]

- .0215***

[- .0374, - .0056]

Indirect effect = - .0157, 95% CI [- .0255, .0086].

Education, Income, Employment and Age as covariables.

- .0958***

[- .1356, - .0560]

Childhood maltreatment severity

Restraint-based cooperation (under surveillance)

Cognitive

Empathy

.0662**

[.0201, .1124]

- .0299***

[- .0447, - .0151]

Indirect effect = - .0063, 95% CI [- .0122, -.0024].

Education, Income, Employment and Sex as covariables.

Childhood maltreatment severity

Third Party Cooperation

Affective

Resonance

.1516 ***

[.0973, .2059]

- .1979***

[- .2352, - .1605]

- .0086

[- .0266, .0093]

Indirect effect = - .0300, 95% CI [- .0455, -.0198].

Education, Income, Employment and Sex as covariables.

Childhood maltreatment severity

Third Party Cooperation

Affective

Dissonance

- .3118***

[- .3641, - .2596]

.0927***

[.0558, .1296]

- .0099

[- .0279, .0080]

Indirect effect = - .0289, 95% CI [- .0476, -.0171].

Education, Income, Employment and Sex as covariables.

*Note.* * *p* < .05; ** *p* < .01; *** *p* < .001. Grey dashed lines represent relations that are not significant. N = 257

**Supplementary Figure 4** Results from the Simple Mediation Analyses on 2PP-ASP

Childhood maltreatment severity

Antisocial Punishment

Self-related Mentalization

- .0395***

[- .0589, - .0201]

- .1160***

[- .1540, - .0781]

.0100**

[.0040, .0161]

Indirect effect = .0046, 95% CI [.0018, .0081]

Education level; Income; Age as covariates

Childhood maltreatment severity

Antisocial Punishment

Cognitive

Empathy

- .0179

[- .0368, - .0010]

- .0823**

[- .1223, - .0423]

.0135***

[.0075, .0194]

Indirect effect = .0015, 95% CI [- .0002, .0036]

Education level; Income; Sex as covariates

Childhood maltreatment severity

Antisocial Punishment

Affective

Resonance

- .0823**

[- .1223, - .0423]

- .0437**

[- .0627, - .0247]

- .0067*

[.0000, .0133]

Indirect effect = .0084, 95% CI [.0046, .0131]

Education level; Income; Sex; Employment as covariates

- .2921***

[- .3468, - .2373]

Childhood maltreatment severity

Antisocial Punishment

Affective

Dissonance

- .0427**

[- .0557, - .0297]

.0024

[- .0042, - .0089]

Indirect effect = .0125, 95% CI [ -.0042, .0089]

Education level; Income; Sex; Age; Employment as covariates

*Note.* * *p* < .05; ** *p* < .01; *** *p* < .001. Grey dashed lines represent relations that are not significant. N = 228

*General instructions for social decision-making tasks (Peysakhovich et al., 2014)*

PLEASE READ THESE INSTRUCTIONS CAREFULLY! Thank you for participating in our study.

We are interested in how people make decisions that affect the payoffs of themselves and other people. During the course of this study, you can earn up to $4.00 USD.

In this study, you will be asked to take part in a series of interactions. In each interaction you will be matched with one or more other people recruited from Mechanical Turk. Each interaction will have you matched with different people. The payoffs for each interaction will be given in points, every point will be worth 2.5 cents at the end of the study - so an interaction for 100 points has $2.50 USD at stake. The rules for each interaction will be different, so please read the instructions carefully!

Some interactions will have different "roles" for different participants. We will ask you what you would like to do in each possible role.

We don't want what happens in one interaction to affect your decisions in another. So at the end of the study we will randomly choose one interaction and use its outcomes to determine your bonus payment. If that interaction had multiple roles, we'll assign you to a role randomly.

Thus, because only one interaction will count, but you don't know which one it will be, it is in your best interest to treat each decision as if it is the only one that matters for your final payoffs.

We're interested in your choices, and we're not interested in "tricking" you in any way. If you see anything out of place or if something doesn't make sense, please let us know by e-mail as it is most likely a bug in the software.

When you are ready for the first interaction, click NEXT to continue.

*Trust Game*

In this interaction you are matched with one other brand new person.

One of you will be person A, one of you will be person B.

Both of you start with 50 points.

First person A makes a choice, then person B responds.

- - - 1. Person A can choose to transfer their 50 points or not.

If person A transfers 50 points the nit is TRIPLED and given to person B (so person B now has 200 points).

- - - 1. Person B can then choose how many of the points they want to transfer back to person A (between 0 and 150).

The graphic below shows a summary of the interaction :

**A makes a choice.**

Person A

Person B

Person A

Person B

***Transfer amount is tripled***

**B makes a choice.**

***Person B can transfer points back***

You must answer this question correctly to be eligible for a bonus if this interaction is picked.

What happens if Person A transfers 50 points and Person B transfers back 25 points ?

- Person A earns 25 points, person B earns 175 points
- Person A earns 50 points, person B earns 50 points
- Person A earns 100 points, person B earns 100 points

If you are person A, do you want to transfer your 50 points to B?

- No transfer
- Transfer 50 points

If you are person B and person A transfers you 50 points (which is multiplied to 150), how many points do you want to transfer back to A? [0 to 150]

*Public Goods Game*

In this interaction you are matched with three other brand new people.

All of you start with 100 points.

Each of you will choose how much of this 100 points to contribute to a group project, and how much to keep for yourself.

All individuals decide at the same time.

1. Everyone chooses how many points to contribute.
2. All contributed points are doubled and split evenly among the group.

Thus, for every 2 points you contribute, you only receive one point from the project. So contributing more points always increases your group’s total payoff, but you always end up with more money if you contribute less.

The graphic below shows a summary of the interaction :

***Each person contributes and contributed points are doubled and split.***

You

Other Person

Other Person

Other Person

You must answer this question correctly to be eligible for a bonus if this interaction is picked.

What contribution BY YOU maximizes payoffs for the GROUP ? What about for yourself ?

- Contributing 100 maximizes payoffs for both the group and myself
- Contributing 0 maximizes payoffs for both the group and myself
- Contributing 100 maximizes payoffs for the group but contributing 0 maximizes my own payoff

How many points do you wich to contribute to the group project ? [0 to 100]

*Ultimatum Game*

In this interaction you are matched with one other brand new person.

One of you will be person A, one of you will be person B.

Person A starts with 100 points and person B starts with o.

First person A makes a choice, then person B responds.

1. Person A will make an offer on how to split the 100 points with person B.
2. Person B will either accept or reject this offer. If person B accepts, then B will get the offered amount and A will keep the rest. If B rejects the offer then both individuals will get 0 points.

The graphic below shows a summary of the interaction :

Person A

Person B

Both get 0

B gets offer

***A makes offer to B***

***Accept***

***Reject***

You must answer this question correctly to be eligible for a bonus if this interaction is picked.

What happens if person B accepts an offer of 20 points ? What happens if person B rejects this offer ?

- If B accepts this offer then A gets 80 and B gets 20, if B rejects then A gets 80 and B gets 0.
- If B accepts this offert than A gets 0 and B gets 0, if B rejects then A gets 0 and B gets 0.
- If B accepts this offer then A gets 80 and B gets 20, if B rejects then both get 0.

If you are person A, what amount will you offer to person B ? [0 to 50]

If you are person B, please use the slider below to indicate your minimum acceptable offer. That is, if the offer that A gives you is below this, you would reject and if the offer A gives you is above or equal to this, you would accept. [0 to 50]

*Dictator Game*

In this interaction you are matched with one other brand new person.

One of you will be person A, one of you will be person B.

Person A starts with 100 points and person B starts with 0.

This interaction has one single decision :

1. Person A will choose how many of the 100 points to transfer to person B.
2. Person B will get the number of points A transfers and A will get to keep the rest.

The graphic below shows a summary of the interaction :

Person A

Person B

***A transfers to B***

You must answer this question correctly to be eligible for a bonus if this interaction is picked.

What happens if person A transfers 20 points ?

- A keeps 80 points and B gets 20 points.
- Both get 20 points.

If you are person A in the interaction, how much will you transfer to person B ? [0 to 50]

*Third Party Punishment Game*

In this interaction you are matched with two other brand new people.

One of you will be person A, one of you will be person B, one of you will be person C.

All individuals start with 100 points.

First person A makes a choice, then person C responds.

1. Person A can choose to take or not take from person B.
2. If A takes, person B loses 50 points and A gets 30 points.
3. If A takes, then C can choose to remove points from A. C can remove up to 100 points from A.

C must pay 1 point for every 5 points they remove from A. Person B is passive in this interaction and makes no decision.

The graphic below shows a summary of the interaction :

***A chooses to take***

***from B***

Person A

Person B

Person C

***If A takes, C can***

***remove points from A***

You must answer this question correctly to be eligible for a bonus if this interaction is picked.

When can C remove points from A ?

- C can only choose to remove points from A if A chooses to take.
- C can always remove points from A.
- C can never remov epoints from A.

If you are person A, will you take from person B ?

- Don’t Take
- Take

If you are person C, how many points will you remove from person A if they take ?

Reminder: if A chooses to take, B loses 50 points and A gains 30 points.

*Second Party Punishment Game*

In this interaction you are matched with one other brand new person.

One of you will be person A, one of you will be person B. All individuals start with 100 points.

This interaction has two parts, in each part both people choose at the same time.

The transfer phase :

1. First, both people will choose to transfer 30 of their points to the other person or not.

Any points transferred are doubled and given to the other person.

The penalty phase :

1. Based on the other person’s transfer decision, each person then chooses wether they want to remove up to 70 points from that other person.

For every 5 points someone wants to remove from the other person, they must pay 1 point.

The graphic below shows a summary of the interaction :

**Transfer phase**

You

Other Person

***Transfer choice***

**Penalty phase**

You

Other Person

***Both people can remove points from each other depending on transfer actions***

You must answer this question correctly to be eligible for a bonus if this interaction is picked.

If person B transfers and A doesn’t, how many points will they each have at the end of the transfer phase ?

- A will have 100 (100 start – 0 transferred), B will have 130 (100 start – 30 transferred + 60 gotten).
- A will have 160 (100 start + 60 gotten – 0 transferred), B will have 70 (100 start – 30 transferred).
- Both will have 130.

Will you transfer 30 points to the other person ?

- Transfer
- Don’t transfer

Depending on if the other person transfers, please use the sliders below to tell us how many points you would like to remove from them :

If the other DOES TRANSFER, I will remove...

If the other DOESN’T TRANSFER, I will remove…

*All Pay Auction*

In this interaction you are matched with one other brand new person.

One of you will be person A, one of you will be person B. All individuals strat with 100 points.

Both people choose at the same time :

There will be a prize worth 100 points.

Each person will choose how many of their 100 points they will spend to try to win the prize.

The person who spends the most points will win the prize (and so get an extra 100 points).

Both people will keep all points that they chose not to spend, and lose any points they spend (wether or not they win).

The graphic below shows a summary of the interaction :

***Both choose how much to spend to try to win prize***

Person A

Person B

You must answer this question correctly to be eligible for a bonus if this interaction is picked.

If person A spends 50 points to try to win and person B spends 30 points, how many points do they each earn ?

- A gets 150 (100 start – 50 spent + 100 for prize), B gets 70 (100 start – 30 spent).
- Both get 100.
- A gets 180 (100 start – 30 spent + 100 for prize), B gets 100 (100 start)

How many points do you want to spend to try and get the 100 point prize?
